# Stage-specific variations in urinary and salt iodine among pregnant women in Beijing

**DOI:** 10.3389/fnut.2026.1728553

**Published:** 2026-03-11

**Authors:** Zhilin Wu, Yubin Zhang, Chao He, Wenzeng Zhang

**Affiliations:** Beijing Shunyi District Center for Disease Control and Prevention, Beijing, China

**Keywords:** gestational stages, iodine deficiency, pregnant women, salt iodine, urinary iodine

## Abstract

**Background:**

Iodine is essential for fetal neural development and thyroid function in pregnant women. Although China has a policy of iodizing salt, salt intake alone may be insufficient to fulfill the iodine needs of pregnant women, particularly in early gestation.

**Purpose:**

To evaluate trimester-specific and annual fluctuations in urine and salt iodine levels among pregnant women in Beijing from 2021 to 2024.

**Methods:**

Iodine data from 400 women were analyzed using descriptive statistics, ANOVA and MEM.

**Results:**

The median urinary iodine decreased from 151.3 μg/L in 2022 to 122.9 μg/L in 2024, falling outside the WHO recommended threshold of 150 μg/L. ANOVA indicated a substantially reduced urine iodine level in the first trimester compared to subsequent trimesters (*F* = 4.72, *P* = 0.011). MEM showed reduced levels in the first trimester (β = –0.34, *P* = 0.01), whereas salt iodine shown no variations across trimesters (β = 0.45, *P* = 0.80). The iodine content in salt remained consistent (19–22 mg/kg), however urine iodine decreased by 17%–24% over time.

**Conclusion:**

Urinary iodine levels among Beijing pregnant women have declined significantly, particularly in early pregnancy. Iodized salt alone is insufficient to meet their needs, therefore enhanced iodine supplementation and early monitoring are crucial to ensuring sufficient iodine intake for embryonic development.

## 1 Introduction

In recent years, iodine intake and nutritional health of pregnant women have received increasing attention. Iodine is an important micronutrient essential for the synthesis of thyroid hormones and is crucial for fetal neural development. During pregnancy, the demand for iodine increases significantly due to increased synthesis of maternal thyroid hormones, increased renal iodine excretion, and the need for iodine for fetal development. Insufficient iodine intake during pregnancy can lead to maternal and infant health problems, such as fetal neural development disorders, premature birth, and thyroid abnormalities. Despite significant progress in iodine nutrition globally in recent decades, iodine deficiency remains a global public health problem. The World Health Organization (WHO) recommends that a median urinary iodine concentration (UIC) of 150–249 μg/L during pregnancy indicates adequate iodine intake. Nevertheless, even in areas where iodine intake is acceptable in the general population, the problem of insufficient iodine intake among pregnant women persists globally. A systematic study of 14,042 pregnant women from different regions showed that the iodine deficiency rate ranged from 16.1% to 84.0% (1). Pregnant women have significantly higher iodine requirements than the general population because the development of the fetal nervous system depends on an adequate supply of maternal thyroid hormones. Data from the United States indicate that iodine deficiency in pregnant women persists, particularly among those with limited iodized salt intake, vegetarian diets, or specific racial and ethnic characteristics. Similarly, studies in China have shown that despite the long standing policy of universal salt iodization, a significant proportion of pregnant women still have urinary iodine concentrations below the World Health Organization’s recommended threshold of 150 micrograms per liter, especially in coastal and urban areas (2). These findings suggest that iodized salt alone may not be sufficient to meet the increased iodine requirements during pregnancy (3–5).

Iodine requirements vary significantly at different stages of pregnancy (6, 7). In early pregnancy, the fetal thyroid gland is not yet fully developed, and fetal development depends entirely on maternal thyroid hormones (7). Therefore, adequate iodine intake in early pregnancy is crucial (8). Previous studies have shown that iodine deficiency in early pregnancy is associated with lower birth weight and increased risk of preterm birth, suggesting that early prenatal iodine deficiency may significantly affect fetal development. A clinical trial involving 212 pregnant women showed that insufficient iodine intake before 14 weeks of gestation can negatively impact fetal brain development and may lead to long term cognitive and language impairments in offspring (9). Although the fetal thyroid gland begins to function in the mid-to-late stages of pregnancy, maternal iodine requirements remain high due to continued fetal development and increased iodine excretion. Insufficient iodine intake in the mid-to-late stages of pregnancy is associated with fetal growth restriction, adverse pregnancy outcomes, and maternal hypothyroidism (10).

Given these concerns, many countries have prioritized iodine supplementation strategies for pregnant women, particularly in areas susceptible to iodine deficiency (11–13). There is evidence that iodine supplementation or iodine rich diets may help improve iodine nutrition in iodine deficient areas and reduce adverse pregnancy outcomes (10). For example, a study in China showed that iodized oil supplementation during pregnancy significantly increased fetal head circumference and reduced the incidence of birth defects from 27% to 11% (*P* = 0.006) (14). Similarly, a randomized controlled trial showed that infants born to mothers who did not receive iodine supplementation had 40% larger thyroid volume and a higher incidence of thyroid hyperplasia compared to infants born to mothers who received iodine supplementation (15). Iodine deficiency is more prevalent in areas with low environmental iodine levels, such as hilly or flood prone areas with low groundwater and soil iodine concentrations (1). In China, areas with groundwater iodine levels below 10 μg/L are designated as iodine deficient areas. This survey was conducted in a district in northeastern Beijing, where previous monitoring studies have consistently shown low iodine concentrations in soil and water (2, 16, 17). Despite the comprehensive salt reform implemented in Beijing in 2017, there is evidence that iodine deficiency persists in pregnant women. A single center cohort study conducted in Beijing from 2017 to 2019 showed that iodine deficiency persisted in pregnant women, especially in early pregnancy, despite the implementation of the salt reform policy (18). Similar results have been recorded in other major Chinese cities, suggesting that pregnant women remain at high risk of iodine deficiency despite the vigorous promotion of iodized salt (19).

Current information suggests that while universal salt iodization has significantly improved iodine nutrition levels in the population, it may not be sufficient to meet the higher iodine requirements during pregnancy and the specific iodine needs at different stages of pregnancy. Continuous assessment of iodine levels and development of appropriate iodine supplementation programs are crucial, especially in early pregnancy. This study aims to assess the annual trends (2021–2024) of urinary iodine concentration (UIC) and household salt iodine content among pregnant women in Beijing, as well as changes across different stages of pregnancy. This study also aims to identify critical periods of iodine deficiency during pregnancy by simultaneously analyzing urinary iodine and salt iodine levels, thereby providing a basis for targeted public health interventions.

### 1.1 Study population

This study was executed as a recurring annual cross sectional monitoring program among pregnant women in a specific administrative area of Beijing, adhering to China’s national Iodine Deficiency Disorders (IDD) surveillance protocol, from 2021 to 2024. The monitoring structure was established to guarantee temporal comparability and geographic representativeness throughout successive years. Annually, five townships were selected by a stratified geographic selection method, with one township designated from each cardinal direction of the district (central, east, west, south, and north). Annual township selection was conducted using the district level administrative registry to identify potential regional variability in iodine exposure. Pregnant women accessing standard prenatal care at community health centers were recruited within each selected township during the annual surveillance period. A stratified sample method based on gestational stage was utilized to guarantee equitable representation of women in the first (1–13 weeks), second (14–27 weeks), and third trimesters (≥ 28 weeks) (20). The goal sample size was established at 100 pregnant women annually, in alignment with the minimum yearly sample size advised in China’s national IDD surveillance standards for specific population categories. The predetermined annual sample size was chosen to guarantee the statistical stability of estimates and the comparability of iodine status indicators across survey years. Recruitment each year persisted throughout the designated observation period until the goal number was achieved.

Eligibility criteria included residence in the study district for at least one year prior to enrollment. Women were excluded if they reported a prior diagnosis of thyroid disease or current use of thyroid related medications, in order to minimize confounding from underlying thyroid dysfunction or pharmacological iodine exposure. All participants provided written informed consent before participation. For each participant, a single spot midstream urine sample of at least 20 mL was collected in a screw capped polypropylene tube; a 10 mL aliquot was used for laboratory iodine determination. In addition, approximately 50 g of household cooking salt was collected from each participant’s home (minimum 50 g required for analysis) in a light protected container. Urine samples were transported under cold chain conditions and stored at 4 °C prior to analysis, while salt samples were stored at room temperature in the dark until testing.

### 1.2 Variables

This study primarily examines the following variables: gestational age, urinary iodine, salt iodine (samples of salt taken from pregnant women’s homes), methods of iodine supplementation [such as the use of qualified iodized salt, iodine containing vitamin supplements, high iodine food consumption (such as seafood) at least twice a month, and iodine preparations], and final dosage.

#### 1.2.1 Urinary iodine

One of the most prevalent methods for determining the iodine nutritional status of a population is urinary iodine testing. The urine iodine concentration is a method that the WHO suggests for tracking iodine insufficiency in the general population. The WHO recommends the use of the population median urine iodine level to assess iodine nutritional status (21). Because pregnant women have increased iodine requirements and because urinary iodine testing can quickly indicate whether there is a risk of iodine deficiency, this approach is especially appropriate for high risk groups (22). With a detection limit of 2.0 μg/L, 2.5 ml urine samples were chosen for this study (23). The amount of iodine that was recovered was 98.6%. Participation in the internal and external quality assurance programs carried out by the Chinese Center for Disease Control and Prevention was observed by all the iodine testing facilities. All the samples were evaluated at the China National Reference Laboratory for their iodine concentration. Every subject had a random 10 milliliter (ml) urine sample taken from midstream. In addition to providing 50–100 g of household salt, each participant also completed a short survey that asked for details such as when they were born, what week they were pregnant, and whether they took iodine supplements. Before enrolling any participants, we obtained written informed consent. The ethics review board at the nearby research facility approved all the participant procedures.

#### 1.2.2 Salt iodine

Household salt iodine concentration was determined using the sodium thiosulfate titration method in accordance with the Chinese national standard GB/T 13025.7-2012. The method’s analytical precision was 2 ppm, satisfying national standards for iodized salt monitoring (24). The UIC was ascertained by arsenic cerium catalytic spectrophotometry (WS/T 107.1-2016) (25). Household salt samples were categorized into four classifications based on measured iodine content (ppm): non-iodized (< 5 ppm), poorly iodized (5–17.9 ppm), adequately iodized (18–33 ppm), and excessively iodized (> 33 ppm), in accordance with national and international monitoring standards.

#### 1.2.3 Quality assurance and quality control (QA/QC)

Both urinary iodine determination and salt iodine analysis were performed according to established operating procedures and followed the same quality assurance and quality control protocols as urinary iodine testing. Each analytical batch included standardized reagents and laboratory quality control samples, and 5% of the samples were randomly selected for repeat testing to assess the reliability of the analysis. External quality assurance was achieved through participation in a proficiency testing program organized by the Chinese Center for Disease Control and Prevention, thereby ensuring the consistency and accuracy of salt iodine determination.

### 1.3 Data analyses

Continuous data (e.g., gestational age, UIC, and household salt iodine) were summarized using mean ± standard deviation (SD) for nearly normally distributed variables and median with interquartile range (IQR) for skewed variables. Categorical variables (e.g., utilization of iodized salt and iodine supplementation) were expressed as counts and percentages. Gestational age was classified into three stages: first trimester (1–13 weeks), second trimester (14–27 weeks), and third trimester (≥ 28 weeks). Annual variations in UIC and household salt iodine levels were initially evaluated using analysis of variance (ANOVA). Prior to parametric analyses, the distribution of UIC was evaluated for normality using graphical inspection (histograms and Q–Q plots) and formal normality testing. When deviations from normality were observed, Box–Cox transformation was applied to UIC to better satisfy the assumptions of parametric testing. To examine gestational stage–specific changes in iodine status while accounting for between individual variability, a mixed effects model (MEM) was constructed. In this model, gestational stage was included as a fixed effect, and participant identity was specified as a random intercept to account for intraindividual correlation. Annual variation was assessed through stratified analyses by survey year to preserve the repeated cross sectional design. Model fit was evaluated using the Akaike information criterion (AIC) and Bayesian information criterion (BIC), with lower values indicating better relative model fit. Adjusted R squared (AjR^2^) was calculated as a descriptive measure of the proportion of variance explained by the fixed effects for model comparison purposes (26, 27). All statistical analyses were conducted using R software (version 3.5.1), and a two sided *P*-value < 0.05 was considered statistically significant.

## 2 Results

### 2.1 Sample characteristics

Between 2021 and 2024, the study encompassed 400 pregnant women, with 100 participants recruited annually. Table 1 summarizes basic demographic data, iodine related behaviors, urinary iodine concentration (UIC), and household salt iodine content by survey year. The average age of participants varied from 30.76 ± 3.54 years in 2021 to 32.07 ± 4.34 years in 2023, with no significant variations across the years. The mean gestational age at sampling was similar across years, roughly 21 to 22 weeks. The proportion of women reporting iodine supplementation increased over time, reaching 100% in 2024. In contrast, the use of non-iodized salt increased from 7.0% in 2021 and 6.0% in 2022 to 25.0% in 2024. Patterns of iodine intake sources also varied by year, with a gradual shift from reliance on iodized salt toward greater use of iodine containing vitamin supplements in later years. The mean household salt iodine concentration increased from 19.17 ± 6.22 mg/kg in 2021 to 22.38 ± 6.94 mg/kg in 2023, followed by a decline to 15.89 ± 9.38 mg/kg in 2024. The median UIC increased from 136.0 μg/L (IQR: 84.2–186.7) in 2021 to 151.3 μg/L (95.3–186.1) in 2022, declined to 117.8 μg/L (71.9–223.7) in 2023, and remained below the WHO recommended threshold in 2024 (122.9 μg/L; 75.6–169.5). Overall, both UIC and household salt iodine content exhibited year-to-year variability across the study period, with lower UIC levels observed in 2023 and 2024 compared with earlier years (Table 1).

**TABLE 1:** Descriptive characteristics of pregnant women by survey year (2021–2024).

Variables	Year			
	2021 (*n* = 100)	2022 (*n* = 100)	2023 (*n* = 100)	2024 (*n* = 100)
Age	30.76 ± 3.54	31.16 ± 4.36	32.07 ± 4.34	31.46 ± 4.06
Gestational age (w)	21.38 ± 9.57	21.86 ± 9.04	22.36 ± 9.46	21.58 ± 8.95
Salt iodine (mg/kg)	19.17 ± 6.22	21.32 ± 6.39	22.38 ± 6.94	15.89 ± 9.38
Urinary iodine (μg/L)	136.0 (84.2, 186.7)	151.3 (95.3, 186.1)	117.8 (71.9, 223.7)	122.9 (75.6, 169.5)
**Usage of iodized salt (%)**				
Yes	93 (93%)	94 (94%)	91 (91%)	75 (75%)
No	7 (7%)	6 (6%)	9 (9%)	25 (25%)
**Iodine supplementation (%)**				
Yes	96 (96%)	93 (93%)	94 (94%)	100 (100%)
No	4 (4%)	7 (7%)	6 (6%)	0 (0%)

Continuous variables (such as gestational age, urinary iodine and salt iodine) are described via statistics, including the interquartile range, median, standard deviation (SD), and mean. For categorical variables, the use of iodized salt, iodine supplementation, frequency and percentage were used for counting.

### 2.2 Variations in salt iodine and UICs among pregnant women in Beijing (2021–2024)

This study conducted descriptive analyses and ANOVA to examine annual variations in UIC and household salt iodine among pregnant women in Beijing from 2021 to 2024. Urinary iodine data were log transformed using the Box–Cox method when necessary to meet normality assumptions. Descriptive statistics revealed significant annual fluctuation in UIC. The median UIC rose from 2021 to 2022, significantly decreased in 2023, and stayed beneath the WHO recommended level in 2024. ANOVA confirmed significant differences in UIC across survey years (*F* = 5.23, *P* = 0.003). In contrast, household salt iodine concentration increased from 2021 to 2023 and declined in 2024; however, no statistically significant differences were detected across years by ANOVA (*F* = 0.45, *P* = 0.72).

### 2.3 Variations in the iodine and salt contents of urine across gestational weeks

The participants in this study were categorized according to their gestational age: first trimester (first thirteen weeks), second trimester (fourteen to twenty seven weeks), and third trimester (thirty eight weeks and beyond). The urine iodine and salt iodine levels of pregnant women were first examined via analysis of variance (ANOVA) across various gestational stages. The changes in these levels were subsequently assessed and compared via the MEM model.

#### 2.3.1 Results of ANOVA at different gestational ages

There was no statistically significant variation in the logarithm of the converted urine iodine levels between different gestational stages in 2024, according to the results of the ANOVA (*P* = 0.45) as shown in Table 2. The amount of salt iodine did not vary significantly across the various stages of gestation (*F* = 0.30, *P* = 0.74). The logarithmic transformed urine iodine levels significantly varied with gestational phase in 2023 (*F* = 4.72, *P* = 0.01). It can be inferred from these findings that the amount of urine iodine varies significantly throughout the first, second, and third trimesters of 2023. The amount of salt iodine did not vary significantly across the various stages of gestation (*F* = 0.12, *P* = 0.89). In 2022, gestational time did not significantly affect urine iodine levels (*F* = 2.14, *P* = 0.14). The levels of salt iodine did not significantly differ between the trimesters (*F* = 0.58, *P* = 0.57). Statistical analysis revealed no significant variation in urine iodine levels among trimesters in 2021 (*F* = 2.01, *P* = 0.16). Furthermore, salt iodine levels did not vary significantly across trimesters (*F* = 0.28, *P* = 0.76).

**TABLE 2:** ANOVA results of urinary iodine and household salt iodine by gestational stage and survey year.

Year	Gestational stage	Mean (log UIC) ± SD	*F*	*P*
**(A) Log-transformed UIC by gestational stage.**				
2021	First trimester (1–13 weeks)	4.32 ± 0.72	2.01	0.16
	Second trimester (14–27 weeks)	4.93 ± 0.66		
	Third trimester (≥ 28 weeks)	4.49 ± 0.59		
2022	First trimester (1–13 weeks)	4.38 ± 0.45	2.14	0.14
	Second trimester (14–27 weeks)	4.86 ± 0.81		
	Third trimester (≥ 28 weeks)	5.06 ± 0.92		
2023	First trimester (1–13 weeks)	4.35 ± 0.74	4.72	0.01
	Second trimester (14–27 weeks)	4.83 ± 0.85		
	Third trimester (≥ 28 weeks)	4.93 ± 0.68		
2024	First trimester (1–13 weeks)	4.65 ± 0.46	0.81	0.45
	Second trimester (14–27 weeks)	4.78 ± 0.53		
	Third trimester (≥ 28 weeks)	4.59 ± 0.88		
**Year**	**Gestational stage**	**Mean salt iodine (mg/kg) ± SD**	***F***	***P***
**(B) Household salt iodine concentration by gestational stage.**				
2021	First trimester (1–13 weeks)	17.55 ± 7.09	0.28	0.76
	Second trimester (14–27 weeks)	20.32 ± 9.44		
	Third trimester (≥ 28 weeks)	18.20 ± 6.75		
2022	First trimester (1–13 weeks)	20.33 ± 7.68	0.58	0.57
	Second trimester (14–27 weeks)	17.46 ± 3.61		
	Third trimester (≥ 28 weeks)	19.20 ± 6.03		
2023	First trimester (1–13 weeks)	22.28 ± 7.62	0.12	0.89
	Second trimester (14–27 weeks)	22.82 ± 6.57		
	Third trimester (≥ 28 weeks)	22.03 ± 6.99		
2024	First trimester (1–13 weeks)	16.96 ± 9.21	0.3	0.74
	Second trimester (14–27 weeks)	15.16 ± 9.89		
	Third trimester (≥ 28 weeks)	16.20 ± 8.88		

#### 2.3.2 Analysis results of the mixed effects model (MEM)

Results from the MEM (Table 3) showed that, compared with the first trimester (reference category), log transformed UIC was significantly lower in the second trimester (β = −0.34, *P* = 0.01) and in the third trimester (β = −0.30, *P* = 0.03). In contrast, no significant associations were observed between gestational stage and household salt iodine concentration. Compared with the first trimester, salt iodine levels in the second trimester (β = 1.16, *P* = 0.47) and third trimester (β = 0.45, *P* = 0.80) did not differ significantly.

**TABLE 3:** Analysis results of the MEM model at different gestational ages.

Variables	Gestational week					Fit index		
	First trimester	Second trimester		Third trimester		AIC	BIC	AjR^2^
	Intercept	β	*P*	β	*P*			
Log (urinary iodine)	4.93	−0.34	0.01	−0.30	0.03	12.45	14.32	0.38
Salt iodine	13.50	1.16	0.47	0.45	0.80	745.3	750.10	0.67

## 3 Discussion

This study comprehensively assessed the annual and pregnancy specific variations in iodine nutrition status among pregnant women in Beijing from 2021 to 2024. Using descriptive analysis, analysis of variance and MEM observed significant interannual fluctuations in UIC, while household salt iodine concentration remained relatively stable. These findings suggest that iodine nutrition status during pregnancy is influenced by multiple factors, not just the availability of iodized salt.

The median urinary iodine concentration recorded in this study was frequently below the WHO recommended range for pregnant women (150–249 μg/L), indicating mild to moderate iodine deficiency at the population level (22). This pattern is consistent with findings from studies in multiple regions of China and other countries. In Zhejiang province, after a reduction in iodized salt iodine concentration, the median urinary iodine concentration among pregnant women decreased to approximately 129 μg/L, with over 59% of participants having a urinary iodine concentration below 150 μg/L (2, 18). Similar iodine deficiency has been recorded in coastal areas of China, despite the expected high consumption of iodine rich seafood in these regions (17, 19). Our findings are consistent with existing data, which showed that the environment or geographical distribution of iodine does not guarantee adequate iodine intake during pregnancy. On the contrary, pregnant women in iodine sufficient areas have significantly higher iodine levels. A study in northern Taiwan showed that the median urinary iodine concentration in pregnant women was 225.3 μg/L, indicating adequate iodine intake, with more than 60% of participants reaching the required level (28).

Similarly, studies in the United States have shown a slightly lower rate of iodine deficiency during pregnancy, with approximately 20%–25% of women having insufficient iodine intake (16). These regional differences may reflect differences in dietary habits, iodine fortification programs, and habitual use of iodine enriched supplements during pregnancy. Studies in Europe further confirm the global prevalence of iodine deficiency during pregnancy. In Spain, despite the widespread use of iodized salt, nearly 40% of pregnant women still have urinary iodine concentrations below the acceptable threshold (29). A national assessment in Latvia showed that even after adjusting for urinary creatinine levels, more than 50% of pregnant women still had insufficient iodine intake (5). Data from the United Kingdom indicate that low iodine levels in early pregnancy are associated with decreased cognitive abilities in offspring (30). These data collectively suggest that the iodine nutritional status of pregnant women in Beijing is situated within the context of a persistent, mild iodine deficiency globally.

The annual variation in UIC found in this study is consistent with other data, indicating that urinary iodine excretion is significantly responsive to short term dietary intake, supplement use, and physiological changes during pregnancy (31, 32). Dietary iodine intake, especially from iodized salt and seafood, varies significantly between individuals and across seasons, directly affecting urinary iodine concentration (11, 33). In addition, the irregular use of iodine supplements makes it difficult to ensure a stable daily intake, leading to fluctuations in UIC (3). Physiological factors such as increased renal iodine clearance and increased thyroid hormone synthesis during pregnancy exacerbate these changes (34, 35). Therefore, short term changes in iodine intake or behavior can lead to quantifiable changes in urinary iodine excretion.

Although this study suggests that household salt iodine levels are relatively stable, iodized salt alone may not be sufficient to meet the increased iodine requirements during pregnancy. Recent studies have reached similar conclusions, indicating that while iodized salt is effective at the population level, it may be insufficient for pregnant women without supplemental iodine sources (4, 36). Changes in dietary habits, reduced intake of non-essential salts, and increased use of non-iodized specialty salts may further reduce the role of iodized salt in overall iodine intake (33). These findings highlight concerns that reliance solely on iodized salt may expose vulnerable populations to iodine deficiency. Analyses related to pregnancy stage showed that urinary iodine levels were significantly lower in early pregnancy compared to other stages. This observation is medically reasonable because the fetus is entirely dependent on maternal thyroid hormones in early pregnancy, and maternal iodine requirements increase before many women begin iodine supplementation (14, 15). As pregnancy progresses, increased awareness of iodine, dietary adjustments and the use of supplements may partially alleviate previous iodine deficiency, leading to increased urinary iodine levels in mid to late pregnancy (9). However, iodine deficiency in early pregnancy can have lasting effects because this period coincides with a critical stage of fetal brain development (27, 37). Human studies have shown that even mild iodine deficiency in early pregnancy can negatively impact neuro development. Data from the ALSAC cohort study indicated that infants born to mothers with insufficient iodine levels in early pregnancy exhibited decreased verbal intelligence and reading abilities later in childhood (30). Iodine deficiency in pregnant women is associated with impaired fetal development, an increased risk of pregnancy complications, and altered maternal and infant thyroid function (9, 10). These findings underscore the importance of ensuring adequate iodine intake before or during early pregnancy for public health. Additional recent studies support these findings (38–43).

### 3.1 Limitations

This study has several limitations that must be noted. First, although a standardized monitoring system was used, participants were from only one administrative district in Beijing. Therefore, the results may not be fully applicable to pregnant women in other regions with varying dietary habits, socioeconomic factors, or iodized salt availability. Second, this study used only a single urine sample to assess urinary iodine concentration (UIC). This method is suitable for assessing population iodine nutritional status; however, it is susceptible to intraindividual variability and cannot accurately reflect long term iodine intake or individual iodine nutritional status. Therefore, misclassification at the individual level cannot be ruled out. Third, data on iodine exposure sources, including intake of iodized salt, iodine supplements, and iodine rich foods, were obtained through self-reporting. This study did not obtain complete data on supplement dosage, formulation, frequency of use, duration, and adherence, nor did it analyze and confirm the iodine content of the supplements. These factors may have led to misclassification of exposures and limited the accuracy of assessing iodine intake from specific sources. Finally, this study lacks comprehensive dietary intake data and did not consider potential seasonal variations in iodine intake, including fluctuations in seafood consumption throughout the year. These unquantified factors may have influenced annual fluctuations in urinary iodine concentration.

## 4 Conclusion

The study results indicate that although iodized salt intake is relatively stable, urinary iodine concentrations in pregnant women fluctuate significantly throughout pregnancy and at different reproductive stages. Urinary iodine concentrations in early pregnancy are significantly lower than recommended levels, suggesting potential iodine deficiency, while concentrations increase significantly in mid and late pregnancy. Despite some positive results from Beijing’s iodized salt promotion program, pregnant women may still require additional iodine sources besides iodized salt to meet their iodine needs throughout pregnancy, especially in early pregnancy. Future public health efforts must emphasize effective monitoring and intervention of iodine nutrition status during pregnancy and promote appropriate iodine supplementation to safeguard maternal and infant health.

## Data Availability

The raw data supporting the conclusions of this article will be made available by the authors, without undue reservation.
